# Ni^2+^-Dependent and PsaR-Mediated Regulation of the Virulence Genes *pcpA*, *psaBCA*, and *prtA* in *Streptococcus pneumoniae*

**DOI:** 10.1371/journal.pone.0142839

**Published:** 2015-11-12

**Authors:** Irfan Manzoor, Sulman Shafeeq, Oscar P. Kuipers

**Affiliations:** 1 Department of Molecular Genetics, Groningen Biomolecular Sciences and Biotechnology Institute, University of Groningen, Nijenborgh 7, 9747 AG, Groningen, The Netherlands; 2 Department of Bioinformatics and Biotechnology, Government College University, Faisalabad, Pakistan; 3 Department of Microbiology, Tumor and Cell Biology, Karolinska Institutet, Nobels väg 16, 17177, Stockholm, Sweden; University Medical Center Utrecht, NETHERLANDS

## Abstract

Previous studies have shown that the transcriptional regulator PsaR regulates the expression of the PsaR regulon consisting of genes encoding choline binding protein (PcpA), the extracellular serine protease (PrtA), and the Mn^2+^-uptake system (PsaBCA), in the presence of manganese (Mn^2+^), zinc (Zn^2+^), and cobalt (Co^2+^). In this study, we explore the Ni^2+^-dependent regulation of the PsaR regulon. We have demonstrated by qRT-PCR analysis, metal accumulation assays, β-galactosidase assays, and electrophoretic mobility shift assays that an elevated concentration of Ni^2+^ leads to strong induction of the PsaR regulon. Our ICP-MS data show that the Ni^2+^-dependent expression of the PsaR regulon is directly linked to high, cell-associated, concentration of Ni^2+^, which reduces the cell-associated concentration of Mn^2+^. *In vitro* studies with the purified PsaR protein showed that Ni^2+^ diminishes the Mn^2+^-dependent interaction of PsaR to the promoter regions of its target genes, confirming an opposite effect of Mn^2+^ and Ni^2+^ in the regulation of the PsaR regulon. Additionally, the Ni^2+^-dependent role of PsaR in the regulation of the PsaR regulon was studied by transcriptome analysis.

## Introduction

*Streptococcus pneumoniae*, an encapsulated bacterium is a common cause of otitis media, bacterial meningitis, bacteremia, and pneumoniae, leading to millions of death every year, particularly in developing countries [1–3]. Although, *S*. *pneumoniae* has an asymptomatic association within the human nasopharyngeal cavity [4], it has also the ability to spread to other sites in the human body to cause severe infections [5–7]. The survival of *S*. *pneumoniae* in different niches inside the human body might depend on the availability of macro- and micro- nutrients on the respective infection sites. Metal ions are an integral part of nutrients, and play a vital role in the regulation of many cellular processes in *S*. *pneumoniae* [8–10]. The deprivation or excess of metal ions may result in impaired growth of bacterial cells [11]. Therefore, proper regulation of metal homeostasis is important for the survival of *S*. *pneumoniae*. For this purpose, *S*. *pneumoniae* possesses metal uptake and -efflux systems that are specific to different metal ions, including manganese (Mn^2+^), zinc (Zn^2+^), copper (Cu^2+^), and iron (Fe^2+^) [12–16]. These systems are tightly regulated by different transcriptional regulators in the presence of specific metal ions [9,10,16–18]. For example, the expression of the *adc* operon encoding Zn^2+^ transporters is repressed by transcriptional regulator AdcR in the presence of Zn^2+^ [10,19]. The *cop* operon, encoding proteins for Cu^2+^ homeostasis is activated by transcriptional regulator CopY in the presence of Cu^2+^ [16]. The expression of Mn^2+^ uptake system *psaBCA* is regulated by transcriptional regulator PsaR and is dependent on the balance between Mn^2+^, Zn^2+^, and cobalt (Co^2+^) [20,21].

The interference or competition of metal ions for metal-sensory proteins has been reported for many bacteria, including *S*. *pneumoniae* [9,16,21–25]. The interplay of metal ions on specific protein depends on the concentration of metal ions, the nature of the coordinating ligands [26–28], and the effect of the Irving-William stability series (where the order is Mn^2+^ < Fe^2+^ < Co^2+^ < Ni^2+^ < Cu^2+^ > Zn^2+^) on protein metal ion affinity [29]. In *S*. *pneumoniae*, the CopY-mediated expression of the Cu^2+^-efflux system depends on the availability of Cu^2+^ and Zn^2+^, where the Cu^2+^ induced expression of Cu^2+^-efflux system is nullified by the addition of high Zn^2+^ concentrations [16]. Similarly, the expression of the PsaR regulon (*pcpA*, *psaBCA*, and *prtA*) is repressed by Mn^2+^ and derepressed by Zn^2+^ [9]. In our previous study, we have demonstrated the opposite effect of Mn^2+^ and Co^2+^ in the regulation of the PsaR regulon [21]. Moreover, metal ions can also compete to bind with extracellular proteins, which ultimately results in the impaired homeostasis of other metal ions. In *S*. *pneumoniae*, Zn^2+^ and Cd^2+^ have been shown to cause intracellular Mn^2+^ deficiency [30,31]. Several proteins with ligase activity have been reported to bind with nickel (Ni^2+^) [32]. However, the role of Ni^2+^ in pneumococcal metabolism and virulence has not been determined.

Here, we used qRT-PCR, β-galactosidase assays, EMSAs, and ICP-MS analyses to investigate the role of Ni^2+^ in the regulation of the PsaR regulon in *S*. *pneumoniae* D39. Our results demonstrate that the expression of the PsaR regulon is highly derepressed in the presence of Ni^2+^ and that a high concentration of Ni^2+^ causes cell-associated Mn^2+^ deficiency in *S*. *pneumoniae*. Furthermore, an opposite effect of Mn^2+^ and Ni^2+^ on the PsaR-mediated expression of the PsaR regulon is found.

## Material and Methods

### Bacterial strains, growth conditions and DNA manipulation

All the bacterial strains and plasmids used in this study are listed in Table 1. *S*. *pneumoniae* D39 was grown in 1% Chelex 100 resin (Bio-rad)-treated Chemically Defined Medium (CDMchelex). Salts of metal ions, *i*.*e*. MnSO_4_ and NiSO_4_ were added separately as specified in the Results section. *Escherichia coli* strain EC1000 was cultured at 37°C. The following concentrations of antibiotics were used in the media for the selection of strains where necessary: tetracycline: 2.5 μg ml^-1^ for *S*. *pneumoniae*; chloramphenicol: 4 μg.ml^-1^ for *Lactococcus lactis*; and ampicillin; 100 μg.ml^-1^ for *E*. *coli*. Chromosomal DNA of *S*. *pneumoniae* D39 was used as a template for PCR amplification [33,34]. All bacterial strains used in this study were stored at -80°C in 10% (v/v) glycerol stock. Primers used in this study are based on the genome sequence of *S*. *pneumoniae* D39 and listed in Table 2.

**Table 1:** List of strains and plasmids used in this study.

Strain/plasmid	Description	Source
***S*. *pneumoniae***		
D39	Serotype 2 strain, *cps 2*	Laboratory of P. Hermans
RW100	D39 Δ*psaR*	[9]
RW104	D39*nisRK* Δ*bgaA*::P*prtA-lacZ*; Erm^R^	[9]
RW109	D39*nisRK* Δ*psaR* Δ*bgaA*::P*prtA-lacZ*; Erm^R^	[9]
IM402	D39 Δ*bgaA*::P*psaB-lacZ*; Tet^R^	[21]
IM403	D39 Δ*bgaA*::P*pcpA-lacZ*; Tet^R^	[21]
IM451	RW100 Δ*bgaA*::P*psaB-lacZ*; Tet^R^	[21]
IM452	RW100 Δ*bgaA*::P*pcpA-lacZ*; Tet^R^	[21]
***E*. *coli***		
EC1000	Km^R^; MC1000 derivative carrying a single copy of the pWV1 *repA* gene in *glgB*	Laboratory collection
***L*. *lactis***		
*NZ9000*	MG1363 Δ*pepN*::*nisRK*	[39]

**Table 2:** List of primers used in this study.

Name	Nucleotide Sequence (5’→3’)
**Primers for qRT-PCR**	
prtA-F	GCAGCCTATGCCCCTAATG
prtA-R	GTTTTAGTGTCTATTACAGG
pcpA-F	CCAATCCTAGCAGATACTCC
pcpA-R	GTAGGAATCGTGAATGG
psaB-F	CCTCAGTGTCTCCTACAAAG
psaB-R	GGCAATTCGGTGTAAGG
psaC-F	CCATTTCCTACAAAATGCCTT
psaC-R	TCCAAAGACAATGGCTCC
psaA-F	CTCGTTCTCTTTCTTTCTG
psaA-R	CTTAACGTCTTCAGGAA
gyrA-F	CGAGGCACGTATGAGCAAGA
gyrA-R	GACCAAGGGTTCCCGTTCAT

### β-galactosidase assays

The β-galactosidase assays were performed as described before [35] by using derivatives of *S*. *pneumoniae* D39 grown till mid-exponential phase of growth (OD_600_ = 0.25) in triplicate in CDMchelex at 37°C supplemented with different metal ion concentrations (w/v) as mentioned in the Results section. Standard deviation was calculated from three independent replicates of each sample.

### Quantitative real time (qRT)-PCR experiments

For qRT-PCR, *S*. *pneumoniae* D39 wild-type was grown in CDM with and without the addition of 0.3 mM Ni^2+^ and harvested at mid-exponential growth phase. RNA was isolated as described before [16]. Additionally, RNA was treated with DNase I (RNase-free) (Thermo Fisher Scientific, St. Leon-Rot, Germany) for 60 min at 37°C to remove any DNA contamination. qRT-PCR was performed in triplicates as described before [16]. The transcription level of the target genes was normalized to *gyrA* transcription using the relative expression software tool [36].

### Inductively coupled plasma-mass spectrometry (ICP-MS) analysis

To measure the intracellular concentrations of metal ions, *S*. *pneumoniae* D39 was grown till OD_600_ = 0.2–0.25 in 20 ml CDMchelex supplemented with either 0.02 mM MnSO_4_, 0.02 mM MnSO_4_ + 0.1 mM NiSO_4_, 0.02 mM MnSO_4_ + 0.3 mM NiSO_4_, or 0.02 mM MnSO_4_ + 0.5 mM NiSO_4_. Cell cultures were washed twice with CDMchelex medium and twice with overnight Chelex (Sigma) treated phosphate-buffered saline (PBS) with 1 mM nitrilotriacetic acid. The cell pellets were dried overnight in a Speedvac at room temperature and lysed in 2.5% nitric acid (Ultrapure, Sigma Aldrich) for 10 min at 95°C by vigorous vortexing. ICP-MS analysis on the lysed cell samples were performed as described before [31]. Amounts of metal ions are expressed in the Result section as μg g^-1^ dry weight of cells.

### DNA Microarray Analysis

To observe the impact of the *psaR* deletion on the transcriptome of *S*. *pneumoniae* in the presence of Ni^2+^, *S*. *pneumoniae* D39 wild-type and its isogenic *psaR* mutant (RW100) [9] were grown in two biological replicates in CDMchelex with 0.3 mM of NiSO_4._ (H_2_O)_6_. Cells were harvested at the mid-exponential growth phase. Further experiments were performed essentially as described before [37]. DNA microarray data were analyzed by using the *MicroPrep* software package as described before [38]. To identify differentially expressed genes a Bayesian p-value <0.001 and a fold-change cut-off of ≥ 2 were applied. The DNA microarray data have been deposited to Gene Expression Omnibus (GEO) with accession number GSE73818.

### Purification of Strep-tagged PsaR and Electrophoretic mobility shift assays

The overexpression and purification of C-terminally Strep-tagged PsaR was achieved in *L*. *lactis* NZ9000 essentially as described before [9,39]. Electrophoretic mobility shift assays (EMSAs) were performed essentially as described previously [10]. In short, PCR products of P*pcpA*, P*psaB*, P*prtA*, and P*adcR* were labeled with [γ-^33^P] ATP. EMSAs were carried out in buffer containing 20 mM Tris-HCL (pH 8.0), 5mM MgCl_2_, 8.7% (w/v) glycerol, 62.5 mM KCl. 25 μg/ml bovine serum albumin, 25 μg/ml poly (dI-dC), and 5000 cpm of [γ-^33^P] ATP-labeled PCR product. Reactions were incubated at 30°C for 30 min before loading on gels. Gels were run in 1 M Tris-borate buffer (PH 8.3) at 95 V for 90 min.

## Results

### Ni^2+^-dependent expression of the PsaR regulon in *S*. *pneumoniae*

In a previous study, we have shown that, like Zn^2+^, Co^2+^ also induces the expression of the PsaR regulon, while addition of Mn^2+^ causes repression of the PsaR regulon [21]. The PsaR regulon comprises the *psa* operon (*psaBCA*), encoding Mn^2+^-dependent ABC transporters, *pcpA*, encoding a choline binding protein and *prtA*, encoding a serine protease. In this study, we decided to explore the impact of Ni^2+^ on the expression of the PsaR regulon. To investigate the impact of Ni^2+^ on the expression of the PsaR regulon, cells were grown in CDM with either 0 or 0.3 mM Ni^2+^, and qRT-PCR was performed. qRT-PCR data revealed that the expression of *pcpA*, *psaBCA*, and *prtA* was highly upregulated in the presence of 0.3 mM Ni^2+^ compared to 0 mM Ni^2+^ (Table 3), suggesting the putative role of Ni^2+^ in the regulation of the PsaR regulon.

**Table 3:** The relative expression of *prtA*, *psaB*, *psaC*, *psaA*, and *pcpA* genes was normalized with the housekeeping gene *gyrA*. The log^2^ fold increase is relative to the expression in the D39 wild-type grown in CDMchelex with 0.3 mM Ni^2+^ to that with 0 mM Ni^2+^. Standard deviation of three independent replications is given in parentheses.

Gene tag^a^	Function^b^	Fold Raito
*spd_0558*	Cell wall-associated serine protease PrtA	4.53 (1.22)
*spd_1461*	Manganese ABC transporter, ATP-binding protein, PsaB	2.83 (0.24)
*spd_1462*	Manganese ABC transporter, permease protein, PsaC	3.15 (0.30)
*spd_1463*	Manganese ABC transporter, ATP-binding protein, PsaA	5.88 (1.55)
*spd_1965*	Choline binding protein PcpA	10.53 (3.09)

^a^Gene numbers refer to D39 locus tags.

^b^D39 annotation/TIGR4 annotation. [34,62]

To further verify the role of Ni^2+^ in the regulation of the PsaR regulon in *S*. *pneumoniae*, the D39 wild-type strain containing either P*pcpA-lacZ*, P*psaB-lacZ*, or P*prtA-lacZ* was grown in CDMchelex and CDMchelex-Mn^2+^ (CDMchelex without Mn^2+^) with the addition of 0, 0.1, 0.3 or 0.5 mM Ni^2+^, and β-galactosidase assays were performed. Our β-galactosidase data (Miller Units) revealed that the expression of the P*pcpA-lacZ*, P*psaB-lacZ*, and P*prtA-lacZ* increased significantly with increasing concentrations of Ni^2+^ in CDMchelex and CDMchelex-Mn^2+^ (Table 4). However, the expression of these transcriptional *lacZ*-fusions was much higher in CDMchelex-Mn^2+^ compared to CDMchelex due to the unavailability of Mn^2+^ in CDMchelex-Mn^2+^. This data indicates that the expression of *pcpA*, *psaBCA*, and *prtA* is regulated by Ni^2+^ and in agreement with our qRT-PCR analysis data mentioned above.

**Table 4:** β-galactosidase activity (miller units) of P*pcpA-lacZ*, P*psaB-lacZ*, and P*prtA*-*lacZ* in *S*. *pneumoniae* D39 wild-type and Δ*psaR* (RW100)grown in CDMchelex and CDMchelex-Mn^2+^ supplemented with various concentrations of Ni^2+^ (mM). Standard deviation of three independent replications is given in parentheses, whereas ND stands for not determined. Noteworthy, *lacZ* was fused to the 3’ end of prtA* on the native chromosomal location, using plasmid pOR113. This might explain the lower Miller Units of P*prtA* compared to P*pcpA* and P*psaB*.

β-galactosidase Activity (Miller Units)							
Medium	D39 (wt)			D39 Δ*psaR*		
	P*pcpA*	P*psaB*	PprtA*	P*pcpA*	P*psaB*	PprtA*
**CDMchelex**						
Ni^2+^ [0.0]	29 (7)	68 (8)	0.57 (0.06)	1363 (35)	1290 (30)	2.1 (0.2)
Ni^2+^ [0.1]	48 (4)	118 (11)	0.92 (0.06)	1226 (42)	1230 (40)	2.1 (0.3)
Ni^2+^ [0.3]	73 (6)	218 (20)	1.38 (0.1)	1195 (52)	1220 (24)	2.2 (0.4)
Ni^2+^ [0.5]	101 (8)	419 (15)	1.57 (0.1)	1190 (23)	1202 (55)	2.0 (0.2)
**CDMchelex-Mn** ^**2+**^						
Ni^2+^ [0.0]	84 (10)	565 (40)	0.74 (0.2)	ND	ND	ND
Ni^2+^ [0.1]	160 (12)	628 (43)	1.24 (0.2)	ND	ND	ND
Ni^2+^ [0.3]	360 (36)	873 (50)	1.62 (0.1)	ND	ND	ND
Ni^2+^ [0.5]	571 (30)	1072 (102)	1.87 (0.1)	ND	ND	ND

### PsaR mediates expression of the PsaR regulon in the presence of Ni^2+^

To check, whether the observed Ni^2+^-dependent high expression of the PsaR regulon is mediated by the Mn^2+^/ Zn^2+^/ Co^2+^-responsive transcriptional regulator PsaR, the *psaR* mutant strain (RW100) containing P*pcpA-lacZ*, P*psaB-lacZ*, and P*prtA-lacZ* were grown in CDMchelex with 0, 0.1, 0.3 or 0.5 mM Ni^2+^. The expression of P*pcpA-lacZ*, P*psaB-lacZ*, and P*prtA-lacZ* was highly derepressed in the *psaR* mutant. We did not observe significant difference in the expression of P*pcpA-lacZ*, P*psaB-lacZ*, and P*prtA-lacZ* in the *psaR* mutant strain at different concentrations of Ni^2+^ (Table 4), indicating that PsaR mediates the Ni^2+^-dependent expression of the PsaR regulon.

To analyze the impact of *psaR* deletion on the global gene expression of *S*. *pneumoniae* and find more targets of PsaR in the presence of Ni^2+^, transcriptome of *psaR* mutant strain was compared with *S*. *pneumoniae* D39 wild-type strain grown in CDMchelex with 0.3 mM Ni^2+^. The expression of *psaR* was significantly downregulated, confirming the inactivation of *psaR* in the *psaR* deletion strain. The expression of *pcpA*, *psaBCA*, and *prtA* was highly upregulated in the *psaR* mutant (Table 5). This data further confirms our β-galactosidase data mentioned above indicating Ni^2+^-dependent derepression of the PsaR regulon. We did not find any new target of PsaR in the presence of Ni^2+^. Notably, an operon (*spd_0616-spd_618*) encoding amino acid ABC transporter proteins was downregulated in our transcriptomic analysis, but in our β-galactosidase assay we did not observe any activity of the respective promotor of this operon in the *psaR* mutant (Data not shown here).

**Table 5:** Summary of transcriptome comparison of *S*. *pneumoniae* D39 wild-type strain with Δ*psaR* grown in CDM with 0.3 mM Ni^2+^.

Gene tag^a^	Function^b^	Ratio^c^	P-value
*spd_0616*	Amino acid ABC transporter, ATP-binding protein	-4.40	1.32E-05
*spd_0617*	Amino acid ABC transporter, permease protein	-5.99	7.46E-07
*spd_0618*	Amino acid ABC transporter, permease protein	-6.19	4.27E-07
*spd_0558*	Cell wall-associated serine protease PrtA	3.02	6.65E-05
*spd_1461*	Manganese ABC transporter, ATP-binding protein	2.39	7.47E-05
*spd_1462*	Manganese ABC transporter, permease protein, putative	2.52	1.46E-04
*spd_1450*	Iron-dependent transcriptional regulator (PsaR)	-4.43	7.45E-07
*spd_1632*	Hypothetical protein	-2.22	9.00E-04
*spd_1965*	Choline binding protein PcpA	14.42	2.65E-09

^a^Gene numbers refer to D39 locus tags.

^b^D39 annotation/TIGR4 annotation. [34,62]

^c^Ratios >2.0 or <2.0 (Δ*psaR* / wild-type).

### Opposite effect of Ni^2+^ and Mn^2+^ in the regulation of the PsaR regulon

Previous studies showed that the PsaR-mediated expression of the PsaR regulon depends on the balance between Mn^2+^, Co^2+^ and/ or Zn^2+^ [9,21,31]. In this study, we observed that the expression of the PsaR regulon was highly derepressed in response to various Ni^2+^ concentrations. Therefore, we decided to explore the influence of Ni^2+^ and Mn^2+^ together on the expression of the PsaR regulon. The expression of P*pcpA-lacZ*, P*psaB-lacZ*, and P*prtA-lacZ* in *S*. *pneumoniae* D39 wild-type was measured at different concentrations of Ni^2+^ and Mn^2+^ in CDMchelex and CDMchelex-Mn^2+^ (Table 6). β-galactosidase data (Miller units) showed that high expression of P*pcpA-lacZ*, P*psaB-lacZ*, and P*prtA-lacZ* at 0.1 or 0.3 mM of Ni^2+^ was nullified by the addition of 0.02 or 0.05 mM Mn^2+^ (Table 6). However, Mn^2+^ repression was higher in CDMchelex compared to CDMchelex-Mn^2+^. This might be due to the fact that CDMchelex contains 5–7 μM of Mn^2+^ which is enough to cause the repression of the PsaR regulon [21]. These results suggest that the Mn^2+^-dependent repression of the PsaR regulon is derepressed by the addition of Ni^2+^.

**Table 6:** Expression level (in Miller units) of P*pcpA-lacZ*, P*psaB-lacZ*, and P*prtA-lacZ* in D39 wild-type in CDMchelex and CDMchelex-Mn^2+^ supplemented with different concentrations of Ni^2+^ and Mn^2+^ (mM). Standard deviation of three independent replicates is indicated in bars.

β-galactosidase Activity (Miller Units)			
Medium	D39 (wt)		
	P*pcpA*	P*psaB*	P*prtA*
CDMchelex	29 (3)	72 (9)	0.50 (0.07)
Ni^2+^ [0.1]	32 (4)	99 (10)	0.91 (0.08)
Ni^2+^ [0.3]	66 (6)	200 (28)	1.20 (0.2)
Ni^2+^ [0.1] + Mn^2+^[0.02]	20 (5)	79 (7)	0.55 (0.05)
Ni^2+^ [0.3] + Mn^2+^[0.02]	36 (27)	142 (12)	0.69 (0.1)
Ni^2+^ [0.1] + Mn^2+^[0.05]	20 (5)	79 (7)	0.30 (0.05)
Ni^2+^ [0.3] + Mn^2+^[0.05]	36 (27)	142 (12)	0.35 (0.1)
CDMchelex-Mn^2+^	90 (15)	550 (50)	0.70 (0.2)
Ni^2+^ [0.1]	180 (18)	640 (48)	1.10 (0.2)
Ni^2+^ [0.3]	390 (60)	890 (106)	1.40 (0.1)
Ni^2+^ [0.1] + Mn^2+^[0.02]	100 (10)	450 (70)	0.80 (0.05)
Ni^2+^ [0.3] + Mn^2+^[0.02]	280 (47)	565 (120)	0.90 (0.1)
Ni^2+^ [0.1] + Mn^2+^[0.05]	50 (10)	210 (70)	0.30 (0.05)
Ni^2+^ [0.3] + Mn^2+^[0.05]	120 (47)	335 (120)	0.60 (0.1)

### Ni^2+^ counteracts the Mn^2+^-PsaR interaction with P*pcpA*, P*psaBCA*, and P*prtA*

**Fig 1:**
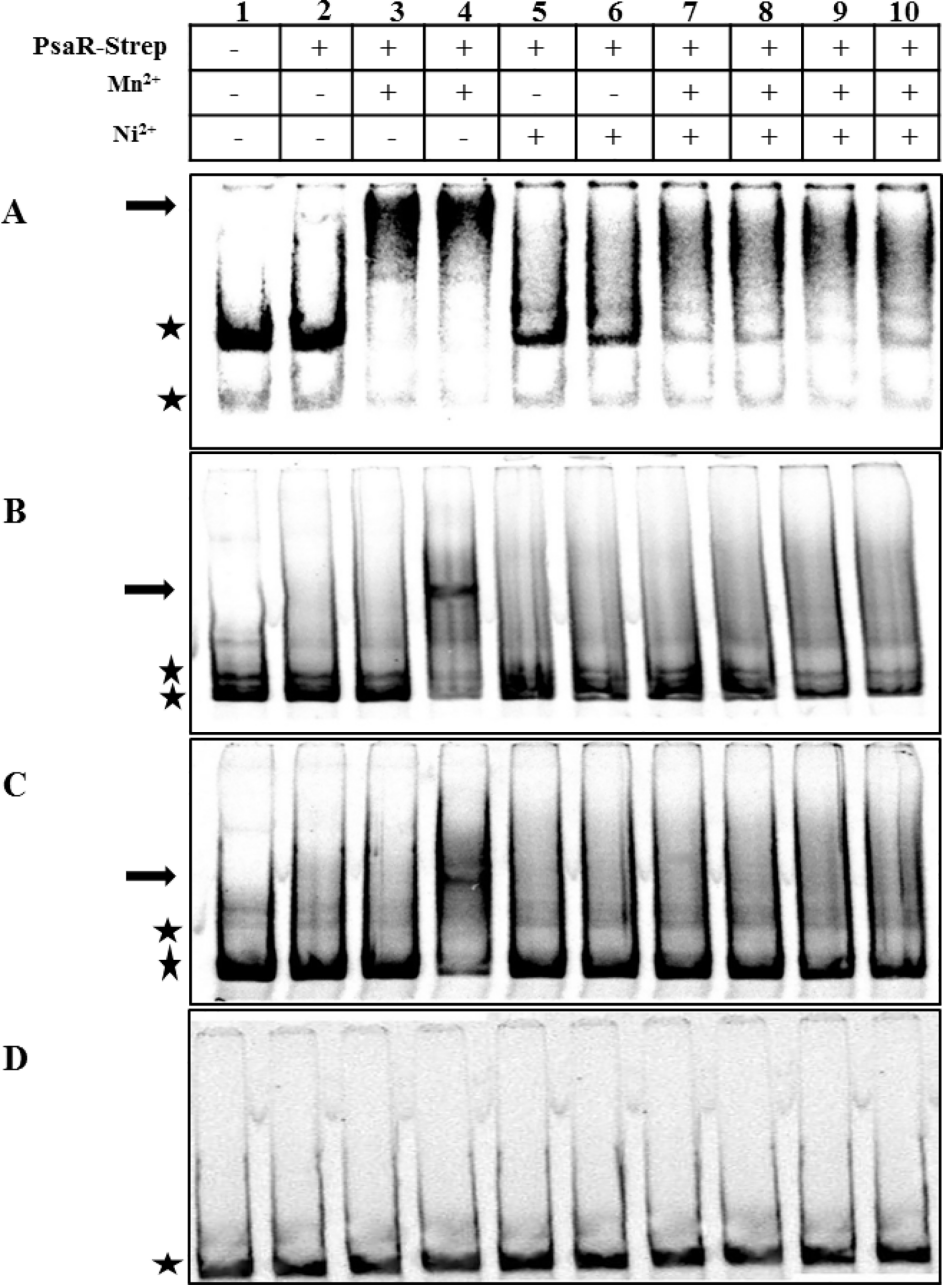
*In vitro* interaction of PsaR-Strep tag with the promoter regions of *pcpA* (A), *psaB* (B), *prtA* (C), and *phtB* (D). PsaR-Strep was added at concentration of 30 nM as indicated above panel, while lane 1 is without added protein. Arrows indicate the position of shifted probe and asterisks indicate the position of free probe. Mn^2+^ was added with concentrations of 0.05 mM in lanes 3, 7, and 9, and 0.1 mM in lane 4, 8, and 10. Ni^2+^ was added with concentrations of 0.2 mM in lanes 5, 7, and 9, and 0.4 mM in lanes 6, 8, and 10.

To find out whether the observed opposite effects of Ni^2+^ and Mn^2+^on the expression of *pcpA*, *psaBCA*, and *prtA* are mediated by the direct DNA binding activity of the PsaR protein, the effects of these metal ions on the binding of PsaR-Strep tag to ^33^P-labeled promoters of *pcpA*, *psaB*, and *prtA* were studied *in vitro*. The promotor region of *phtB* was used as a negative control. Due to the metal-ion chelating ability of EDTA, we decided to exclude it from all buffers used to perform EMSAs. PsaR-Strep tag was not able to bind with the promoter regions of *pcpA*, *psaB*, and *prtA* without the addition of any metal ion (Fig 1A, 1B and 1C. Lane 2) which is in agreement with the previous study [9]. First of all, we checked the DNA binding activity of PsaR-Strep to the promoter regions of *pcpA*, *psaB*, and *prtA* with different concentrations of Mn^2+^. We observed that 0.05 and 0.1 mM Mn^2+^ were able to stimulate the binding of PsaR-Strep tag to the promoter region of *pcpA*. However, only 0.1 mM Mn^2+^ was able to stimulate the binding of PsaR-Strep to the promoter regions of *psaB* and *prtA* (Fig 1A, 1B and 1C. Lane 4). No binding of PsaR-Strep to the promoter regions of *psaB*, and *prtA* was observed at 0.05 mM Mn^2+^ (Fig 1A, 1B and 1C. Lane 3). Interestingly, no shift in the promoter regions of *pcpA*, *psaB*, and *prtA* was observed with 0.2 or 0.4 mM Ni^2+^ (Fig 1A, 1B and 1C. Lane 5 and 6), suggesting that Ni^2+^ does not stimulate the binding of PsaR with *pcpA*, *psaB*, and *prtA* promoters. Previously, it has been shown that Zn^2+^ binds to the PsaR in such a way which leads to the inactivation of Mn^2+^-PsaR interaction with the promoter regions of *pcpA*, *psaB*, and *prtA* [9]. We hypothesized that like Zn^2+^, Ni^2+^ also interferes in the Mn^2+^-dependent binding of PsaR-Strep to the promoter regions of *pcpA*, *psaB*, and *prtA*. Therefore, we decided to explore the influence of Ni^2+^ on the *in vitro* Mn^2+^-PsaR-Strep tag interaction. Interestingly, the binding of PsaR to all three promoters in the presence of Mn^2+^ was impaired with the addition of Ni^2+^ (Fig 1 Lanes 7–10). This data suggests that the Mn^2+^-PsaR interaction with *pcpA*, *psaB*, and *prtA* promoters is competed away in the presence of Ni^2+^, indicating a direct role of Ni^2+^ in the regulation of the PsaR regulon through PsaR.

### A high concentration of Ni^2+^ in the medium leads to Mn^2+^ deficiency in the cells

**Fig 2:**
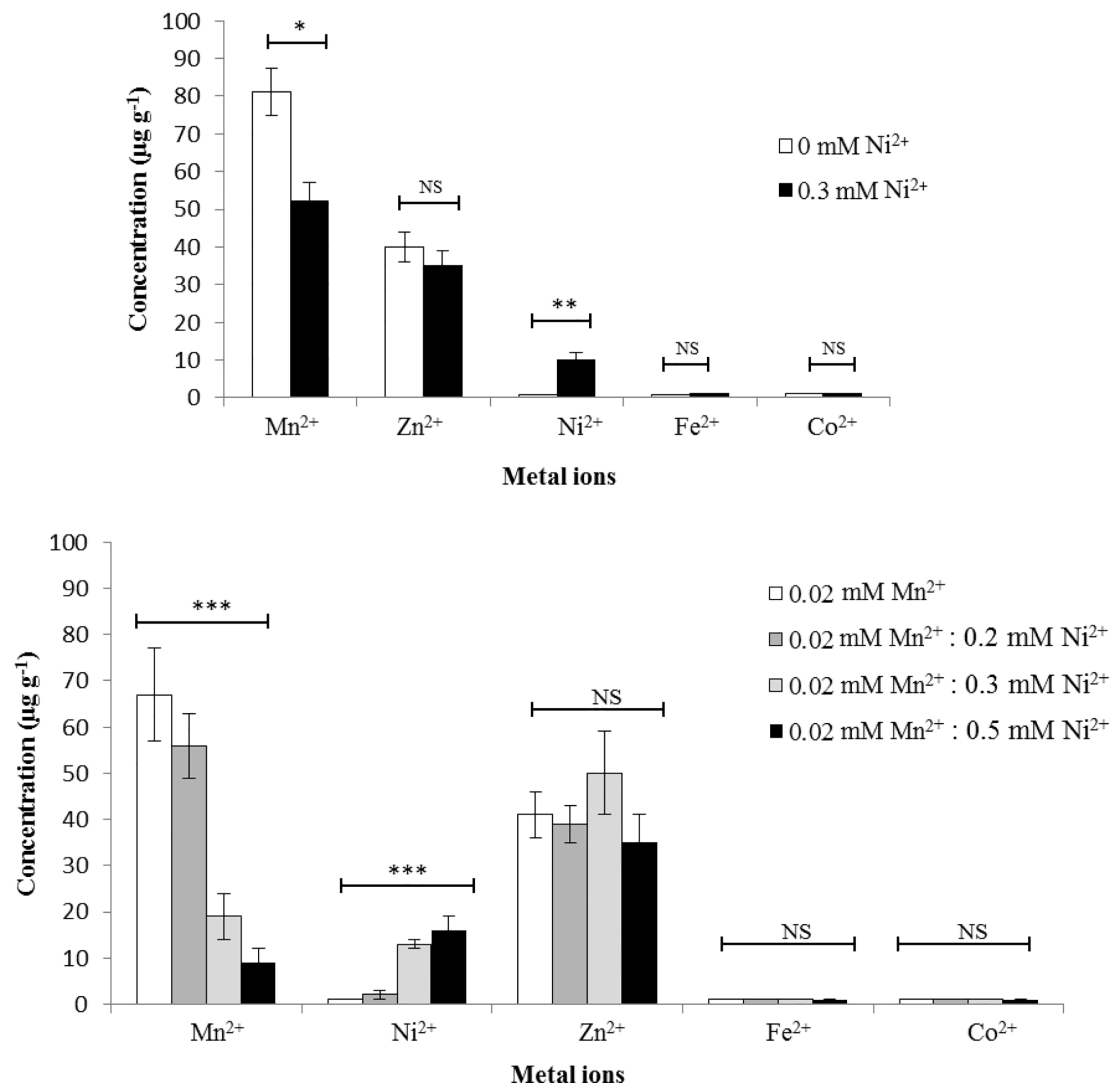
(A) Cell-associated metal ion concentrations (expressed ug g^-1^) of S. pneumoniae D39 wild type when grown in CDMchelex with either 0 mM or 0.3 mM Ni^2+^. (B) Metal ions contents of *S*. *pneumoniae* D39 wild-type when grown in CDMchelex containing 0.02 mM Mn^2+^ with addition of 0, 0.1, 0.3 or 0.5 mM Ni^2+^. The statistical significance of the differences in the mean metal concentrations was determined by one-way ANOVA (NS not significant, *P<0.01, and ***P<0.0001)

To determine the cell-associated concentrations of metal ions, we performed an ICP-MS analysis on the cells grown in CDMchelex either with 0 or 0.3 mM of Ni^2+^. ICP-MS data revealed that the cells grown in the presence of 0.3 mM Ni^2+^ accumulate 10-fold (P<0.01, One way ANOVA) more Ni^2+^ (Fig 2A) compared to cells grown in the absence of Ni^2+^. No significant difference in the concentrations of other metal ions was observed in our ICP-MS analysis except for Mn^2+^. The concentration of Mn^2+^ was reduced by 1.5-fold (P<0.01, One way ANOVA) in the presence of Ni^2+^ (Fig 2A). This data indicates that high concentration of Ni^2+^ leads to Mn^2+^ deficiency in the cell. To study this in more details, we have checked the impact of various concentrations of Ni^2+^ on the cell-associated Mn^2+^. Cells were grown in CDMchelex with the addition of 0.02 mM Mn^2+^, and 0, 0.1, 0.3 or 0.5 mM Ni^2+^. As expected, addition of Ni^2+^ in medium leads to an increased cell-associated Ni^2+^ concentration. The cell-associated Ni^2+^ concentration was increased by 2-fold (P<0.01, One way ANOVA) at 0.1 mM Ni^2+^, 13-fold at 0.3 mM Ni^2+^, and 16-fold at 0.5 mM Ni^2+^ when compared to 0 mM Ni^2+^ (Fig 2B). ICP-MS analyses data further revealed that an increasing concentration of Ni^2+^ leads to a decrease in the concentrations of Mn^2+^. The cell-associated concentration of Mn^2+^ was decreased by 1.25-fold (P<0.01, One way ANOVA) at 0.1 mM Ni^2+^, 3.52-fold (P<0.01, One way ANOVA) at 0.3 mM Ni^2+^, and 7.4-fold (P<0.01, One way ANOVA) at 0.5 mM Ni^2+^ (Fig 2B) when compared to the Mn^2+^ concentration at 0 mM Ni^2+^. Notably, the cell-associated concentration of other metal ions (Zn^2+^, Fe^2+^, and Co^2+^) was not affected (Fig 2). This data demonstrate that Ni^2+^ has ability to cause Mn^2+^ starvation which ultimately leads to the high expression of the PsaR regulon in the presence of Ni^2+^.

## Discussion

Adherence to epithelial cells of human nasopharynx is the primary step of *S*. *pneumoniae* towards the pathogenesis [40]. The pneumococcal surface adhesion protein, PsaA and choline binding protein, PcpA are among those proteins that promote pneumococcal adherence in nasopharyngeal epithelial cells and colonization in mice [14,41,42]. Similarly, PrtA, a serine protease containing an LPXTG-anchor motif, is expressed on the surface of nearly all virulent pneumococcal strains and is required for full virulence in animal models [43,44]. The *pcpA*, *psaBCA*, and *prtA* genes comprise the PsaR regulon and their expression is regulated by transcriptional regulator PsaR [9]. The role of Mn^2+^, Zn^2+^, and Co^2+^ in the regulation of *pcpA*, *psaBCA*, and *prtA* (PsaR regulon) has already been established [9,21,45]. In this study, we investigated the role of Ni^2+^ on the expression of the PsaR regulon. The expression of the PsaR regulon was increased with the increasing concentrations of Ni^2+^ and this increased expression of the PsaR regulon is directly linked with cell-associated Mn^2+^ deficiency caused by a high concentration of Ni^2+^. Moreover, Mn^2+^ and Ni^2+^ have opposite regulatory effects on the expression of the PsaR regulon in *S*. *pneumoniae*. Where, Mn^2+^-binding represses the expression of the PsaR regulon, Ni^2+^ derepresses the repression caused by Mn^2+^.

Mn^2+^ is an important transition metal ion that is a cofactor for many pneumococcal proteins which are involved in the colonization, virulence, and resistance to oxidative stress in *S*. *pneumoniae* [15]. Mn^2+^ accumulation shows significant flexibility and cells can survive even at a 3% concentration of the normal accumulation level [45,46]. *S*. *pneumoniae* has a dedicated system for Mn^2+^ transport (PsaBCA) that consists of two ABC transporters (PsaBC) and a cell surface salute binding protein (PsaA) [47–49]. Previous studies have shown that PsaA is not only important for virulence [14,41], but also has a direct role in the accumulation of cell associated Mn^2+^ [48,49]. PsaA has the ability to bind Zn^2+^ and Mn^2+^ [46,48]. The binding affinity of PsaA to Zn^2+^ is much higher compared to that of Mn^2+^, and PsaA-Zn^2+^ interaction led to the ~40% decrease in cell associated Mn^2+^ accumulation [31,46]. Structural studies of PsaA have revealed that Cd^2+^ can also bind to PsaA and ultimately results in the reduction of cell-associated Mn^2+^ [30]. Recently, it was shown that PsaA can also bind to other *d*-block elements including Ni^2+^ [50]. This unique property of PsaA to bind with different metal ions makes its role very important in the life style of *S*. *pneumoniae*. In our ICP-MS analysis, we observed a cell-associated Mn^2+^ deficiency in the presence of relatively high concentrations of Ni^2+^. Therefore, based on our ICP-MS data, we can speculate that most likely Ni^2+^ interacts with PsaA, which leads to Mn^2+^ deficiency.

Biochemical studies of transcriptional regulator PsaR of *S*. *pneumoniae* showed that PsaR harbors two pairs of metal binding sites where Mn^2+^ or Zn^2+^ can bind [51]. Similarly, Mn^2+^-responsive regulators DtxR from *Corynebacterium diphtheria* and MntR from *Bacillus subtilis*, which are homologous of PsaR, also have two metal binding sites [52,53]. The binding of DtxR to the *tox* operon in *C*. *diphtheria* not only depends on the availability of Mn^2+^ but also on Co^2+^, Fe^2+^, and Ni^2+^ [54]. Similarly, The Mn^2+^-dependent DNA binding activity of MntR in *B*. *subtilis* is diminished in the presence of Ni^2+^, Zn^2+^, and Fe^2+^ [55–57]. The metal responsive transcriptional regulators, ScaR of *Streptococcus gordonii* and SloR of *Streptococcus mutants* also belongs to DtxR family, and are homologous to PsaR [58–60]. Interestingly, the PsaR binding site is similar to the operator sequences of ScaR and SloR [61]. This might suggest that PsaR uses a similar mechanism of metal ion competition for regulatory metal ion homeostasis as other member of DxtR family regulators adopt.

It has been previously demonstrated that PsaR represses the expression of the PsaR regulon in the presence of Mn^2+^ whereas Zn^2+^ and Co^2+^ relieved this repression [21,61]. Moreover, the *in vitro* studies of the interaction of PsaR to its target promotors showed that both Zn^2+^ and Co^2+^ could bind to PsaR in a different way [21]. When Zn^2+^ interacts with PsaR, it relieves the PsaR interaction with the promoter regions of the PsaR regulon, whereas Co^2+^, just like Mn^2+^, stimulates the interaction of PsaR with the promoter regions of the PsaR regulon [9,21]. Here, we demonstrated that Mn^2+^-PsaR interaction leads to the binding of PsaR to the promoter regions of *pcpA*, *psaBCA*, and *prtA* which is an agreement with previous studies [9]. However, the Mn^2+^-PsaR interaction with *pcpA*, *psaB*, and *prtA* promoters was alleviated by the addition of Ni^2+^ which suggests that the observed transcriptional response of the PsaR regulon is directly linked to the interaction of Ni^2+^ and Mn^2+^ on the PsaR-promoter interactions. In conclusion, we have shown that the interaction of PsaR to Ni^2+^ plays a similar role as Zn^2+^, to induce derepression by PsaR in competition with Mn^2+^.
